# Redefining Surgical Materials: Applications of Silk Fibroin in Osteofixation and Fracture Repair

**DOI:** 10.3390/biomimetics9050286

**Published:** 2024-05-11

**Authors:** Jose A. Foppiani, Iulianna C. Taritsa, Lacey Foster, Armaan Patel, Angelica Hernandez Alvarez, Daniela Lee, Gavin J. Lin, Theodore C. Lee, Dominika Gavlasova, Maria J. Escobar-Domingo, David L. Kaplan, Samuel J. Lin

**Affiliations:** 1 Division of Plastic Surgery, Beth Israel Deaconess Medical Center, Harvard Medical School, Boston, MA 02215, USA; josefoppiani@yahoo.co.za (J.A.F.); danielalee@hms.harvard.edu (D.L.);; 2 Keck School of Medicine, University of Southern California, Los Angeles, CA 90033, USA; ldfoster@usc.edu; 3 Department of Biomedical Engineering, Tufts University, Boston, MA 02155, USA; armaanpatel814@yahoo.com (A.P.);; 4 Nobles and Greenough School, Dedham, MA 02026, USA; 5 Georgetown University, Washington, DC 20001, USA; 6 Institute of Clinical and Experimental Medicine, 140 21 Prague, Czech Republic

**Keywords:** silk fibroin, osteofixation, biocompatibility, sustainable biomaterials, translational science

## Abstract

Silk and silk derivatives have emerged as a possible alternative in surgical device development, offering mechanical strength, biocompatibility, and environmental sustainability. Through a systematic review following PRISMA guidelines, this study evaluated silk fibroin’s application across pre-clinical and clinical settings, focusing on its role as screws and plates for osteofixation. A comprehensive search yielded 245 studies, with 33 subjected to full-text review and 15 ultimately included for qualitative analysis. The findings underscore silk fibroin’s superior properties, including its tunable degradation rates and ability to be functionalized with therapeutic agents. In vivo and in vitro studies demonstrated its efficacy in enhancing bone healing, offering improved outcomes in osteofixation, particularly for craniofacial defects. Silk fibroin’s remarkable attributes in biodegradation and drug release capabilities underscore its potential to enhance patient care. Ultimately, silk fibroin’s integration into surgical practices promises a revolution in patient outcomes and environmental sustainability. Its versatility, coupled with the continuous progress in fabrication techniques, signals a promising horizon for its widespread acceptance in the medical field, potentially establishing a new benchmark in surgical treatment. Further research is expected to solidify the transition of silk products from basic science to patient care, paving the way for widespread use in various surgical applications.

## 1. Introduction

Bone fractures represent a prevalent form of injury spanning across all demographic groups [1]. The current paradigm for the treatment of bone fractures incorporates a diverse array of plating techniques, including conventional, dynamic, and locked plating methods [2,3]. These methodologies have been validated for their efficacy in ensuring stability and facilitating the transfer of load across the fractured site [2,3]. Despite these advantages, the application of bone plating is not devoid of potential postoperative complications. Issues such as plate failure and infection not only necessitate additional surgical intervention and morbidity to the patient but also impose a substantial financial burden [2,3]. Furthermore, the permanence of bone plating underscores a significant limitation, as it requires surgical procedures for its removal or replacement, adding another layer of complexity to the patient’s treatment and recovery process [2,3].

The burgeoning interest in silk as a biomaterial for medical applications, particularly in the domain of fracture treatment, underscores a significant advancement in the field of biomedical engineering [4]. Silk fibroin, derived from natural sources, has emerged as a highly versatile material, capable of being fashioned into various forms including films, wafers, and gauzes, making it an invaluable resource for a multitude of medical applications [4,5]. A notable innovation in this realm is the development of silk-based plates and screws for bone fracture management, which represents a departure from traditional metal alloys [6,7].

Silk-based implants for bone repair exhibit remarkable properties, such as superior biocompatibility, biodegradability, and an ability to be tailored to specific anatomical requirements without the need for additional modification procedures [6,7,8]. These implants demonstrate a unique combination of strength and flexibility, allowing for them to be precisely shaped to match the contours of the bone, thus eliminating the challenges associated with the fitting of standard absorbable plates [6,7]. Moreover, the reduced risk of breakage and the ability to maintain structural integrity highlight the material’s potential to enhance the efficacy and safety of bone fracture treatments [6,7]. The utilization of silk in this context not only facilitates a more natural healing process but also paves the way for future innovations in implantable medical devices [9,10,11].

The objective of this systematic review is to comprehensively assess the utilization of silk in both pre-clinical and clinical settings, thereby establishing a foundational basis for translational science. This endeavor aims to bridge the gap between experimental research and clinical practice, facilitating the advancement of innovative treatment modalities.

## 2. Methods

The protocol for this study was registered in advance with PROSPERO under the study number CRD4202342084 [12]. This study was completed following the guidelines outlined in the Preferred Reporting Items for Systematic Reviews and Meta-Analyses (PRISMA) statement [13].

### 2.1. Eligibility Criteria

The inclusion criteria for studies were specified as any research documenting the use of silk or silk-derived materials in bone plates or screws. The complete set of eligibility requirements can be found in Table 1.

### 2.2. Search Strategy

An exhaustive review of the literature employing subject headings, controlled vocabulary, and keywords was conducted to identify studies on PubMed/MEDLINE and Web of Science, covering publications up to January 2024. The detailed methodology of our full-text search is available on PROSPERO.

### 2.3. Study Selection

The search findings were transferred to Covidence, where a dual-phase screening methodology was implemented for the selection of studies. Initially, two reviewers independently assessed the titles and abstracts. In cases of disagreement between the first two reviewers, a third reviewer was engaged to make a final decision on the study’s inclusion or exclusion after a moderating discussion. During the second phase, these same reviewers conducted a comprehensive review of the full texts, selecting studies that met the predetermined eligibility criteria. When disputes occurred between the two initial reviewers, a third was called upon to resolve the inclusion or exclusion of a study, following a process of discussion and moderation.

### 2.4. Data Extraction/Synthesis

The variables extracted were the type of study (in vivo vs. in vitro), focusing on silk-based implants, the population studied (animal vs. human), and the methodology employed to assess implant integration and healing. Additionally, outcomes investigated included biomechanical properties, durability, and therapeutic effectiveness of silk implants, with results indicating their potential advantages over traditional materials.

### 2.5. Quality Assessment

In order to evaluate the potential for bias, the quality assessment tool developed by the National Institute of Health (NIH) was employed. Each study was classified according to its bias risk level as either “low risk”, “moderate risk”, or “high risk” [14].

### 2.6. Statistical Analysis

The wide variety of topics explored in the studies included in this systematic review precluded the possibility of undertaking any form of analysis beyond qualitative synthesis.

## 3. Results

**Figure 1:**
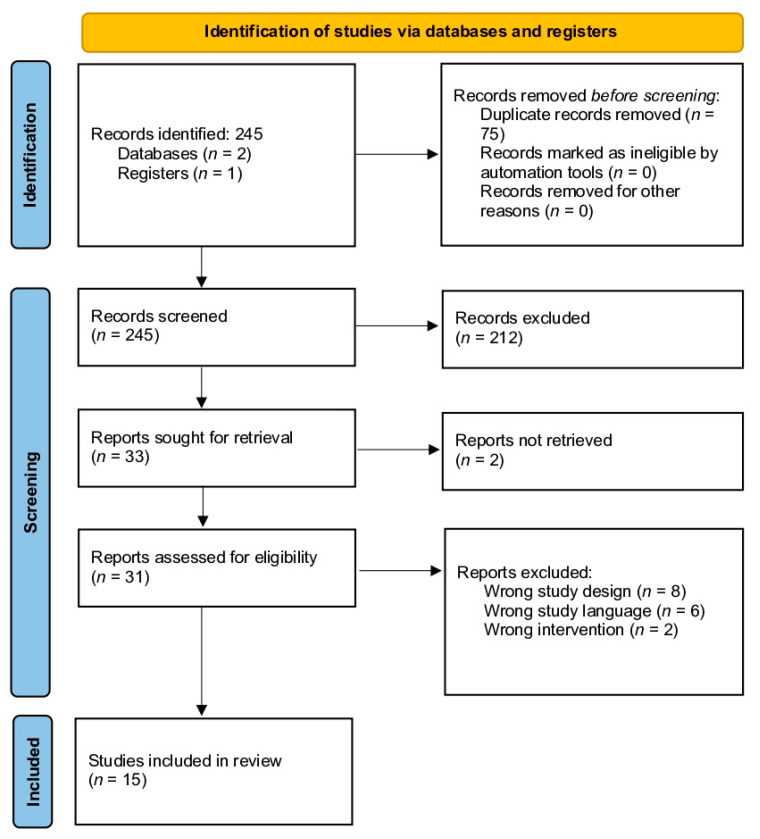
Systematic review and meta-analysis (PRISMA) guideline flow diagram.

The electronic search initially identified 245 silk-related orthopedic/plastic surgery studies, which ultimately yielded 33 studies for full-text reviews (Figure 1). After thorough assessment and subsequent exclusion, 15 studies from 2014 to 2023 were included for quantitative analysis (Table S1) [6,15,16,17,18,19,20,21,22,23,24,25,26,27,28]. These consisted of seven in vitro experimental studies and eight in vivo experimental studies.

### 3.1. In Vitro Studies

In the experimental in vitro studies, silk-based implant coatings, alloy sheets, composite scaffolds, screws, plates, and dental implants were investigated for biocompatibility, antimicrobial properties, mechanical strength, and potential function. A complete summary of protocols and reported outcomes can be found in Table S2.

Elia et al. investigated the development of a silk fibroin-based coating for titanium shins, studs, and dental implants [15,26]. They utilized two methods: electrodeposition directly onto the titanium substrate and melting electrogels followed by dispensing onto titanium to form the coating. Their study revealed that silk coatings could be successfully applied to titanium substrates using the electrogelation approach, demonstrating adequate adhesion and mechanical strength to withstand stresses during implantation. Both the electrogelation coating and electrodeposition processes leverage the unique mechanical and physical properties of silk proteins to adjust adherence, coating thickness, and provide a relatively simple and adaptable system for dental implant functionalization [15,26]. The adhesion strength of silk coatings fell within the range of other biological coatings, with a notable advantage being the ease of application of silk coatings.

After studying two techniques of developing silk-based coatings, Elia et al. investigated the mechanical properties and drug release characteristics of silk protein-based electrodeposited coatings for dental implants, utilizing silk concentrations ranging from 1% to 10% [15,26]. Their findings indicated that the adhesion strength of hydrated silk coatings was influenced by both concentration and surface texture. There was a noticeable trend of increasing strength with higher protein concentrations, aligning with the range of values seen in coatings made from collagen, chitosan, or hydroxyapatite commonly used in implants. Coatings produced from a 10% silk solution and processed for 10 min exhibited comparable strength and thickness to clinically relevant implant coatings [15,26]. Additionally, these coatings served as reservoirs for storing and releasing model compounds, with the amount of drug released showing consistency with other conventional systems.

Given the well-studied poor cell adhesion and mechanical properties of polycaprolactone (PCL) scaffolds, Vyas et al. investigated the enhancement of PCL scaffolds by incorporating a composite of PCL silk microparticles (PCL/SMP) [16]. Their study revealed that the PCL/SMP composite exhibited increased elasticity and shear-thinning behavior compared to the PCL scaffold alone. The inclusion of SMPs strengthened the PCL matrix, thereby improving the mechanical properties of the scaffolds, which were found to be comparable to the lower region of trabecular bone in terms of uniaxial compression mechanical properties [16]. Moreover, scaffolds with higher SMP concentration loading displayed heightened roughness and hydrophobicity, along with notable calcium mineral deposition. The presence of SMPs also accelerated the enzymatic degradation of the scaffolds. Notably, there was a clear distinction in material properties between samples containing SMPs and those comprising PCL alone, with properties generally correlating with increasing SMP content [16]. Initial biocompatibility assessments demonstrated that human adipose-derived stem cells (hADSCs) remained viable and proliferated for up to 21 days on scaffolds containing up to 20 wt% SMP loading [16]. Importantly, the presence of SMPs significantly improved the degradation rate, mechanical properties, and cell proliferation of the scaffolds, indicating that incorporating silk particles can mitigate key drawbacks of PCL.

Shi et al. developed silk screws loaded with gentamicin (GSSs) alongside pure silk screws (PSSs) containing varying concentrations of gentamicin to evaluate their antimicrobial properties, strength, and gentamicin release [20]. They observed no statistically significant difference in the bending strength between the PSSs and GSSs. However, there was a notable increase in the degradation rate of the GSS groups compared to the PSS groups, which was statistically significant [20]. The most effective concentration for inhibiting the growth of S. aureus and E. coli was found to be 16 mg of gentamicin per 1 g of silk, a feature absent in the PSS groups lacking antimicrobial activity [20]. Consequently, GSSs may hold potential for reducing the risk of implant-related infections during the postoperative period.

Yan et al. fabricated silk screws employing different concentrations of silk nanofibrils (SNFs) to assess both biocompatibility and mechanical strength [19]. Their investigation revealed a notable enhancement in screw stiffness upon the incorporation of 10% SNF. Moreover, the 10% SNF variant demonstrated considerable stability under physiological conditions, as evidenced by its diameter expansion and water absorption after 72 h in a PBS solution [19]. Furthermore, there was a significant rise in fluorescence intensity over days 3 to 7, indicating cellular adhesion to human bone marrow mesenchymal cells. This observation suggests favorable metabolic activity and proliferation of cells in the preparation containing 10% SNF [19].

James et al. investigated the local delivery of mi-RNA using silk-based screws and films to assess the inhibition of endogenous expression of osteoinductive antagonists [22]. They observed that incorporating mi-RNAs onto the surface of silk-based screws resulted in a smooth, uniform coating and a burst release profile from silk materials, followed by sustained release over 7 days [22]. Importantly, they found no apparent adverse effects, indicating good cytocompatibility. Moreover, both AS-miR-214-loaded silk films and screws significantly augmented osteogenic differentiation markers and mineralization [22]. This approach offers a means to regulate cellular differentiation and function related to osteogenesis and angiogenesis synergistically. Overall, the study highlights the feasibility of developing silk-based devices to promote enhanced osteogenesis.

Due to the limited application of magnesium-based alloys stemming from their susceptibility to corrosion and degradation, Asadi et al. devised a coating comprising silk fibroin and cellulose nanocrystals (CNCs) for magnesium alloys, aiming to evaluate the biocompatibility and corrosion resistance of the coating [25]. The addition of CNCs to the SF coatings was found to augment the β-sheet content, reaching 31% in the SF-CNC coating. Coatings of SF and SF-CNC on polydopamine (PD)-treated AZ31 magnesium alloy exhibited superior adhesion, with smooth edges and no detachment observed [25]. This composite coating notably enhanced both barrier properties and corrosion protection. Preparing the AZ31 substrate with PD prior to applying the protective coating was deemed essential for achieving significant corrosion protection [25]. Moreover, the SF-CNC coating significantly improved the biocompatibility of AZ31 alloy in terms of cell viability and adhesion/spreading onto human fetal osteoblast cells. In summary, the incorporation of natural CNCs into the SF coating, offering excellent biological properties alongside enhanced anticorrosion features, presents a promising solution for surface modification of magnesium alloys in bio-implant applications [25].

These studies collectively highlight the versatility and potential of silk-based materials in orthopedics, offering solutions for implant coatings, tissue engineering scaffolds, and antimicrobial implants.

### 3.2. In Vivo Studies

In the experimental in vivo studies, silk-based bone clips, screws, and sponges were investigated for biocompatibility, bone regeneration capacity, mechanical strength, and degradation. The animal models used were rats or rabbits. A complete summary of protocols and reported outcomes can be found in Table S3.

Perrone et al. utilized a rat model to examine the biocompatibility, strength, and functionality of silk fibroin screws [6]. Twenty-eight screws were surgically implanted into the femurs of rats following procedures similar to those used for metallic fixation, involving drilling a pilot hole and inserting a self-tapping screw [6]. The silk-based screws were well tolerated by the rats and initiated typical bone remodeling processes without experiencing any failures during implantation. Assessment of swelling indicated no significant concern regarding mechanical integrity loss during implantation, as the material did not absorb a substantial amount of water or alter in diameter until after 8 min in vitro [6]. The rats displayed mobility on all four legs immediately after surgery and on postoperative days 3 and 4. Over the 4- and 8-week periods, the rats continued to exhibit mobility without displaying signs of pain, discomfort, or distress. Two rats experienced wound dehiscence, which resolved with antibiotic treatment. After 4 and 8 weeks, early resorption of the silk screws was observed [6]. However, upon examination, the screws remained securely in place within the bone without any visible adverse reactions. This study illustrated the feasibility of using resorbable silk screws with shear properties comparable to those of resorbable PLGA materials. Notably, the screws were self-tapping, eliminating the need for a two-step implantation process required with current resorbable screws [6].

Koolen et al. conducted a study comparing the use of bone morphogenetic protein-2 (BMP-2)-loaded silk screws to pure silk screws in a rat model to assess their impact on biocompatibility and bone regeneration properties [17]. All 15 animals exhibited mobility on all four legs immediately after the effects of anesthesia subsided post-surgery. There were no indications of pain in any of the animals during examination for 3–4 days after surgery, and no instances of infection or wound dehiscence were observed [17]. During the surgical procedure, the screws came into contact with blood and other bodily fluids but remained intact for insertion. All samples were removed at the designated time points of 1, 3, and 6 months and subjected to histological evaluation. At the 1-month mark, histological sections of BMP-2-loaded implants exhibited the highest levels of new collagen deposition, along with a dense population of osteoclasts and osteoblasts compared to non-loaded samples [17]. Furthermore, the BMP-loaded samples continued to demonstrate increased recruitment of osteoclasts and osteoblasts around the perimeter of the BMP-loaded screws throughout the 6-month study period [17].

Bottagisio et al. investigated the efficacy of vancomycin-enriched silk fibroin sponges (AFN-PSFs) compared to pure silk fibroin sponges (PSFs) in combating methicillin-resistant Staphylococcus epidermidis (MRSE) infection [21]. They examined the local treatment of MRSE-induced nonunion using a well-established rat model known for inducing osteomyelitis and septic nonunion. The study revealed that the AFN-PSF group exhibited nearly complete or complete fracture healing in most samples, accompanied by new bone formation during the remodeling phase and the formation of neoformed bone covering the implant [21]. On days 7 and 14, rats treated with PSF displayed a significant increase in neutrophils compared to both the SHAM and AFN-PSF groups [21]. Particularly, the PSF group exhibited a considerable difference in inflammation compared to both the SHAM and AFN-PSF groups. Analysis using a semi-quantitative histological scoring system indicated a notable difference between the AFN-PSF and PSF groups in terms of total score and inflammatory patterns, with PSF showing higher values associated with poorer bone healing [21]. Overall, AFN-PSF demonstrated sufficient antimicrobial activity to prevent bone infection in vivo, suggesting its potential application for local arthroplasty prophylaxis in patients at high risk of infection.

Suryavanshi et al. developed composite materials consisting of polycaprolactone (PCL) with varying proportions of magnesium oxide (MgO) and silk, which were then fabricated into bone screws [23]. In vitro experiments revealed that both the addition of MgO and silk to PCL significantly enhanced the tensile strength and modulus of the material. Among the MgO–silk–PCL composites, the composition containing 10% MgO, 20% silk, and 70% PCL (referred to as MSP) exhibited notably improved tensile properties, resembling those of soft tissues such as tendons, ligaments, and cancellous bone [23]. Consequently, this MSP composite was selected for the subsequent in vivo investigation. After 2 weeks, moderate granulation tissue was observed at the wound site, but by 8 weeks, there were no discernible incision marks at the surgical site. Serum biochemistry assessments indicated normal functioning of vital organs with no signs of adverse reactions following implantation of the composite bone screw [23]. Importantly, the pull-out strength of the composite implant was significantly higher (1.85 fold) than that of the PCL screw alone [23]. Moreover, the fixation strength value of the MSP composite screw was comparable to accepted values reported in the literature for forces experienced by the native anterior cruciate ligament (ACL) during regular daily activities [23].

Shi et al. evaluated their previously developed gentamicin-loaded silk-based material in a rabbit model, discovering that screws containing 4 mg of gentamicin per 1 g of silk were securely affixed to the femurs and exhibited rough surfaces [20]. These screws made close contact with the bone cortex, and their surfaces were covered with a substantial amount of soft tissue. Pathological analysis at 1 month revealed the presence of multinucleated macrophages, but no clustering of inflammatory cells was observed in either bone or soft tissue. Staining with hematoxylin and eosin (HE) and Masson’s trichrome revealed new bone formation surrounding the screw and hyperplasia [20]. Microcomputed tomography scans conducted at 1, 2, and 3 months indicated that the screws remained well positioned in the femurs without experiencing fracture or detachment, and no apparent adverse reactions were detected in the surrounding tissues [20]. The threads of the screws had become shallower, the screw surfaces appeared rough, and high-density bone substances were observed around the screw, indicating favorable biocompatibility and potential clinical applicability [20].

Zhang et al. developed silk fibroin/gelatin (RSF-G) screws and compared them with pure silk fibroin (RSF) screws in a rat model to evaluate their biocompatibility and strength [24]. They observed preliminary resorption and degradation of both RSF and RSF/G hydrogels at four weeks after implantation, with RSF/G hydrogels showing more degradation at eight weeks compared to RSF hydrogels [24]. At two and four weeks post-subcutaneous implantation, the areas surrounding both RSF and RSF/G implants exhibited infiltration of polymorphonuclear and mononuclear cells, which significantly decreased at eight weeks, indicating good biocompatibility of both hydrogels. The morphologies of RSF and RSF/G screws remained unchanged after eight weeks, with no instances of screw failure, breakage, or loosening, leaving the screw heads above the femurs [24]. The screws demonstrated sufficient strength for insertion into the bone with self-tapping, even in the presence of surrounding blood and saline, suggesting that the limited swelling of the hydrogels did not impede the implantation process and allowed for insertion by surgeons for at least 10 min. X-ray results indicated that screw insertion did not affect knee mobility in the rats [24]. MRI images at two weeks post-implantation revealed a strong signal around the screw heads, indicating tissue edema, which disappeared by eight weeks, demonstrating the biocompatibility of both RSF and RSF/G screws in bone. Additionally, the MRI signal of RSF/G screws was higher than that of RSF screws, suggesting greater water absorption by RSF/G screws due to the high MRI signal of water. This study suggests that RSF/G screws hold significant promise in the field of orthopedics, particularly for bone fixation applications [24].

Yeon et al. developed a 3D-printed bone clip device comprised of polylactic acid (PLA), hydroxyapatite (HA), and silk for internal fixation in a rat model [27]. Their research revealed that the PLA/HA/silk composite bone clip exhibited comparable mechanical properties but demonstrated superior biocompatibility compared to both PLA and PLA/HA clips. In an animal study, the PLA/HA/silk composite bone clip demonstrated excellent alignment of bony segments across femur fracture sites, with the bone clip well positioned [27]. Radiological assessments confirmed the excellent alignment of bony segments across the fracture site with the bone clip properly positioned. This innovative 3D-printed, patient-specific bone clip addresses the limitations of existing internal fixation devices such as nails, plates, and screws, eliminating the need for drilling holes in the bone [27]. This eliminates potential complications such as secondary fractures, bone necrosis, delayed union, and postoperative infections.

Yan et al. devised a novel bulk material based on silk fibroin with a high content of hyaluronic acid (HA) for evaluating fixation strength in a rabbit model [28]. They discovered that both the maximal failure load and stiffness were notably higher in the SF/40% HA–SF group at 4 and 16 weeks post-surgery. Furthermore, the screw exhibited superior osteoinductivity in the SF/40% HA–SF group, correlating positively with surgical outcomes [28]. Compared to rabbit anterior cruciate ligament (ACL) reconstruction fixed with a titanium screw and superior to that fixed with a PLLA screw, the maximal failure load of SF/40% HA–SF in this study is similar. No significant bone loss or tunnel widening was observed in the femoral tunnel of the SF/40% HA–SF group. Radiological examinations of screws in the SF/40% HA–SF group at 12 weeks post-surgery revealed no evident corrosion [28]. Most tendon grafts in the SF/40% HA–SF group were closely adjacent to the bone tunnel, with minimal observation of the graft–bone interface. The distinct four-layer structure of the regenerated tendon–bone interface, comprising bone, calcified fibrocartilage, uncalcified fibrocartilage, and tendon graft, at 12 weeks post-ACL reconstruction in the SF/40% HA–SF group demonstrates its osteoinductivity. Semi-quantitative analysis confirmed that tendon–bone integration in the SF/40% HA–SF group was significantly superior to that in the SF group at both time points [28]. The compressive modulus and yield strength of SF/40% HA–SF approached those of natural compact bone, indicating promising potential for clinical translation of this new bulk material.

In essence, in vitro studies examined silk-based coatings, composite scaffolds, and screws for biocompatibility, mechanical strength, and drug release characteristics, showcasing promising results in terms of adhesion strength, drug release, and mechanical properties. Also, in vivo studies evaluated the biocompatibility, bone regeneration capacity, and mechanical strength of silk-based bone clips, screws, and sponges in animal models, demonstrating favorable outcomes such as successful bone remodeling, antimicrobial activity, and excellent alignment of bony segments. These findings collectively underscore the potential of silk-based materials in orthopedics, offering versatile solutions for implant coatings, tissue engineering scaffolds, antimicrobial implants, and internal fixation devices.

## 4. Discussion

Research surrounding silk fibroin, both as a bulk material and in prepared constructs, has developed significantly in the past several years. The studies identified by this search demonstrate both in vitro and in vivo the refinement and optimization of several characteristics of silk as a biomaterial, including its adhesion strength, mechanical properties (e.g., maximal failure load and stiffness), drug release, osteoinductivity, biocompatibility, and drug release properties. Altogether, the potential for clinical translation of silk constructs to patient care is imminent especially due to the advantages silk has over metals and current resorbables. Silk fibroin’s use in humans may likely be a milestone in surgical device development. The incorporation into the hospital setting may be accelerated due to the manufacturing and safety incentives that silk offers over devices in the current marketplace.

Silk products have multiple manufacturing benefits that make them attractive for mass fabrication. Novel silk processing techniques, such as thermally processed silks, require fewer processing steps and lower production costs compared to original silk fibroin processing methods [29]. Silk bulk materials have another benefit of being renewable and sustainable to mass produce. Their manufacturing process does not require the use of harsh chemicals or crosslinkers [30,31]. Silk is also environment friendly in its overall low global carbon footprint, which has been demonstrated in studies on the topic [32,33]. Current options on the market for internal fracture fixation, such as metal plates, screws, and bone clips, are associated with a great deal of environmental detriment [34,35]. Silk manufacturing has great reproducibility, which, combined with its lower cost, predisposes silk-based devices to favor well in a global market, especially in lower-resource settings.

Silk fibroin constructs represent a favorable alternative to metals for surgical applications due to its biodegradability characteristics as well. Biodegradability, the property of being able to gradually break down as mediated by specific biological activity in vivo, allows for implants made from silk to let treated tissue return to its native state [36]. Silk is able to degrade over a tunable amount of time, in the range from minutes to years, depending on the patients’ needs. Advances in silk device fabrication now allow for more customizable materials variables, porosity, and silk secondary structure, all of which grant the ability to change silk’s degradation time [37,38]. Controlling degradation rate is a useful feature for multiple surgical devices, and especially for fracture fixation where gradual degradation can allow for better bone healing and remove the need for a second surgery to remove the device [6].

The advances described by the studies identified in our review also showcase a significant and unique advantage of silk over other materials: its ability to be functionalized. Manufacturing silk products with certain coatings or as a drug-laden part that exudes the drug agent over time, gives a novel dimension of being able to combine therapeutic agents with silk devices. Functionalizing silk with antibiotics, for instance, may be able to prevent infection and prevent the necessity for future hospital procedures related to the implant. Certain materials being used in surgical devices, such as current resorbables and metals, are associated with high rates of infection [39,40,41,42]. Thus, functionalized silk would have clear benefits of being used over other materials.

With the growing number of advances in silk manufacturing, as demonstrated by the in vitro and in vivo studies identified in our review, the barriers to implementing silk in the clinical setting are disappearing. Silk-based coatings, tissue engineering scaffolds, antimicrobial implants, and internal fixation devices have an increasing number of advantages to current options on the market. Silk-based osteofixation systems for craniofacial fractures may be the first area where silk is used in the clinical setting, due to the numerous mechanical and biodegradable advantages silk has over metal and current resorbable fixation devices. We anticipate silk’s great potential in the global market. Future studies will likely build on the current research presented here to further accelerate silk’s adoption into patient care.

## 5. Limitation

This study represents an aggregation of the current literature concerning silk fibroin applications for implants and screws, yet it is important to acknowledge certain constraints that could impact the breadth of our conclusions. A primary limitation is the absence of aggregate data across studies, which, combined with the heterogeneity of the research methodologies, sample sizes, and application contexts, precludes a comprehensive statistical analysis. This diversity reflects the broad potential of silk fibroin but also complicates the synthesis of findings into a unified framework for clinical application. Accordingly, while this review illuminates the potential benefits and uses of silk fibroin, it underscores an imperative for transitioning fundamental scientific findings into clinical research. This transition is essential to validate and leverage silk fibroin’s advantages in practical healthcare applications.

## 6. Conclusions

The culmination of research into silk fibroin as a biomaterial has significantly advanced the field of surgical device development. Recent studies underscore the comprehensive benefits of silk, from its enhanced mechanical properties and biocompatibility to its superior environmental footprint compared to traditional materials like metals. The inherent advantages of silk fibroin, including its tunable degradation rates and the capability for functionalization with therapeutic agents, position it as a groundbreaking alternative for a range of surgical applications. Particularly, the potential for silk-based osteofixation systems in craniofacial surgeries highlights the imminent clinical translation of silk constructs. The shift towards silk fibroin in patient care not only promises to improve outcomes but also aligns with sustainability goals, offering a dual advantage in the face of growing environmental concerns. As manufacturing processes for silk become more streamlined and cost-effective, the material’s adoption in lower-resource settings appears increasingly feasible. Future research is expected to further validate the clinical efficacy of silk fibroin, paving the way for its widespread incorporation into healthcare practices worldwide.

## Figures and Tables

**Table 1:** Inclusion and exclusion criteria.

Inclusion Criteria	Exclusion Criteria
Studies focused on silk screws or screws utilizing silk derivatives.	Literature reviews, as they provide secondary analysis rather than original research data.
Studies centered on silk plates or plates using silk derivatives.	Systematic reviews, which aggregate findings from multiple studies but do not offer new experimental data.
Clinical studies investigating the application, efficacy, or safety of silk-derived screws and plates.	Letters to the editor, which often include preliminary observations, comments, or critiques without detailed empirical evidence.
Experimental studies exploring the properties, biocompatibility, or innovative uses of silk in surgery.	Studies focusing on other silk products not directly related to screws or plates, to maintain the research focus’s specificity.

## Data Availability

Data sharing not applicable. No new data were created or analyzed in this study. Data sharing is not applicable to this article. All information relevant to this systematic review is part of the manuscript, figures, tables, and/or digital supplemental content. Additional information can be found within the publicly available PROSPERO protocol for this study. If any further information is required, the reader may contact the corresponding author for clarifications.

## Supplementary Materials

The following supporting information can be downloaded at: https://www.mdpi.com/article/10.3390/biomimetics9050286/s1, Table S1: Study characteristic summary; Table S2: In vitro study results; Table S3: In vivo study results.
