# Supplementary material for: Redefining Surgical Materials: Applications of Silk Fibroin in Osteofixation and Fracture Repair
Source: Biomimetics (Basel). 2024 May 11;9(5):286. doi: 10.3390/biomimetics9050286 (PMC11118403; doi:10.3390/biomimetics9050286)
Supplement: Supplementary file 1 [file biomimetics-09-00286-s001.zip › biomimetics-2922023-supplementary.pdf]

Table S1: Study Characteristic Summary

Title	First Author, Year	Type of Study	Intervention/ Device	Model Organism	Medical Specialty Affiliation
The Use of Silk-Based Devices for Fracture Fixation	Perrone, 2014	In Vivo	Silk Fibroin Screws	Sprague- Dawley Rats	Plastic and Reconstructive Surgery, Otolaryngology
Silk Electro Gel Coatings for Titanium Dental Implants	Elia, 2014	In Vitro	Silk Fibroin Dental Coatings	Titanium Screws and Studs	Basic Science
3D Printing of Silk Microparticle Reinforced Polycaprolactone (PCL) Scaffolds for Tissue Engineering Applications	Vyas, 2022	In Vitro	Composite Silk Fibroin-PCL Scaffolds	Human Adipose-Derived Mesenchymal Cells	Basic Science
Increased Osteoid Formation in BMP-2-Loaded Silk-Based Screw	Koolen, 2016	In Vivo	Silk Fibroin Screws	Sprague- Dawley Rats	Plastic and Reconstructive Surgery
An Antibacterial and Absorbable Silk-Based Fixation Material with Impressive Mechanical Properties and Biocompatibility	Shi, 2016	In Vitro	Silk Fibroin Screws	Gram positive staphylococcus aureus and Gram negative Escherichia coli (E. coli) cells	Orthopedic Surgery
Controllable Production of Natural Silk Nanofibrils for Reinforcing Silk-Based Orthopedic Screws	Yan, 2023	In Vitro	Silk Fibroin Screws	Human Bone Marrow Mesenchymal Stem Cells	Orthopedic Surgery
Degradation of Internal Fixation Materials Based on Antibacterial and Absorbable Silk Containing Different Gentamicin Concentrations	Shi, 2022	In Vivo	Silk Fibroin Screws	New Zealand Rabbits	Basic Science
Vancomycin- Nanofunctionalized Peptide-Enriched Silk Fibroin to Prevent Methicillin-Resistant Staphylococcus epidermidis-Induced Femoral Nonunions in Rats	Bottagisio, 2022	In Vivo	Silk Fibroin Sponges	Wistar Rats	Orthopedic Surgery
Silk Biomaterials-Mediated miRNA Functionalized Orthopedic Devices	James, 2019	In Vitro	Silk Fibroin Screws, Plates and Silk	Human Umbilical Vein Endothelial Cells, Human Mesenchymal Stem Cells	Basic Science
Development of Bone Screw Using Novel Biodegradable Composite Orthopedic Biomaterial: from Material Design to In Vitro Biomechanical and In Vivo Biocompatibility Evaluation	Suryavanshi, 2019	In Vivo	Silk Fibroin Screws	Sprague-Dawley Rats	Basic Science
Robust Bioactive Protein-Based Screws with Dual Crosslinked Network for Internal Bone Fixation	Zhang, 2022	In Vivo	Silk Fibroin Screws	Sprague- Dawley Rats	Orthopedic Surgery
Cellulose Nanocrystal Reinforced Silk Fibroin Coating for Enhanced Corrosion Protection and Biocompatibility of Mg-Based Alloys for Orthopedic Implant Applications	Asadi, 2021	In Vitro	Silk Fibroin Alloy Sheets	Fetal Osteoblast Cells	Basic Science

Electrodeposited Silk Coatings for Bone Implants	Elia, 2014	In Vitro	Silk Fibroin Dental Implants	Titanium Studs	Oral and Maxillofacial Surgery
New Concept of 3D Printed Bone Clip (Polylactic acid/Hydroxyapatite/Silk composite) for Internal Fixation of Bone Fractures	Yeon, 2017	In Vivo	Silk Fibroin Bone Clip	Sprague- Dawley Rats	Otolaryngology
An Interference Screw Made Using a Silk Fibroin-Based Bulk Material with High Content of Hydroxyapatite for Anterior Cruciate Ligament Reconstruction in a Rabbit Model.	Yan, 2021	In Vivo	Silk Fibroin Screws	New Zealand Rabbits	Sports Medicine

Table S2: *In Vitro* study results

Study	Protocol	Outcomes	NIH Quality Assessment
Elia, 2014	In the first technique, silk was electrodeposited directly onto titanium substrates, while the second technique utilized melted electrogels dispensed onto titanium to form coatings. Both techniques were evaluated for coating uniformity using stylus profilometer and dial thickness gauge. Mechanical strength of coated titanium studs was assessed using a universal mechanical testing machine.	The silk fibroin coatings for titanium implants demonstrated adequate adhesion and mechanical strength, highlighting the potential for functionalizing dental implants.	Low Risk of Bias
Vyas, 2022	PCL/SMP composites were prepared via melt blending, utilized for scaffold fabrication using extrusion-based additive manufacturing, and assessed for in vitro biological compatibility using human adipose-derived stem cells maintained in MesenPRO RS™ media under standard conditions.	Silk microparticles enhanced the polycaprolactone (PCL) scaffolds by improving mechanical properties and biocompatibility, offering promise for bone tissue engineering.	Low Risk of Bias
Shi, 2016	Silk screw blank molds were prepared using wax, filled with HFIP silk solution, subjected to methanol and water gradients, immersed in distilled water, dried, and baked, with resulting silk blanks cut into slices and screws; additionally, various concentrations of silk-based screws were prepared, including pure silk-based screws and those with different concentrations of GSS.	Silk screws loaded with gentamicin demonstrated antimicrobial properties that could reduce postoperative infections.	Low Risk of Bias
Yan, 2023	Dialyzed SNF hydrogels were dried to obtain silk rods, which were then processed into silk screws and characterized, with varying concentrations of SNFs represented accordingly; additionally, commercial human bone mesenchymal stem cells isolated from a healthy individual were utilized for cell culture experiments.	Silk screws fabricated with silk nanofibrils demonstrated enhanced stiffness and biocompatibility suggesting their potential for orthopedic applications.	Low Risk of Bias
James, 2019	AS-miR-214 silk devices, prepared using surface coating, were used in various tests to examine in vitro biocompatibility and inhibition of osteoinductive antagonists.	Incorporating miRNAs onto silk-based screws led to a smooth, uniform coating and a burst release profile followed by sustained release over 7 days, with no apparent adverse effects, significantly augmenting osteogenic differentiation markers and mineralization, indicating good cytocompatibility and suggesting a feasible approach for developing	Low Risk of Bias

		silk-based devices to promote enhanced osteogenesis.	
Asadi, 2021	Electrochemical and in vitro corrosion studies were conducted in Hank's solution to simulate physiological conditions, employing electrochemical techniques such as open circuit potential (OCP), potentiodynamic polarization (PP), and electrochemical impedance spectroscopy (EIS), with subsequent morphological analysis by SEM. Cytocompatibility assessments involved cell culture with fetal osteoblast cells to evaluate cell viability and adhesion using cell viability assays and fluorescence microscopy to observe cell morphology and adhesion on the samples.	Composite silk fibroin and cellulose nanocrystal coating for magnesium alloys enhanced corrosion resistance and biocompatibility, which could improve the performance of orthopedic implants made from these materials.	Moderate Risk of Bias
Elia, 2014	An aqueous silk fibroin solution was electrogelled onto titanium studs using a platinum wire or titanium stud, followed by characterization of the titanium stud surface using stylus profilometry and SEM, and mechanical analysis through uniaxial tensile and shear tests. Additionally, hydroxyapatite and glycerol were incorporated into the silk solution prior to electrogelation, and water and methanol annealing were performed before mechanical testing, with gel deposition quantified and compound incorporation and release assessed using fluorescein isothiocyanate-dextran samples.	The adhesion strength of silk protein-based electrodeposited coatings for dental implants was influenced by concentration and surface texture, with higher concentrations exhibiting increased strength comparable to clinically relevant coatings, while also serving as reservoirs for storing and releasing model compounds with consistency akin to conventional systems.	Low Risk of Bias

Table S3: *In Vivo* study results

Study	Protocol	Outcomes	NIH Quality Assessment
Perrone, 2014	Silk screws were implanted identically to current metallic fixation procedures where a pilot hole is drilled and a self-tapping screw inserted.	Demonstrated the biocompatibility and functionality of silk fibroin screws in a rat model showing successful implantation without failures, typical bone remodeling processes, and early resorption without adverse reactions, suggesting the feasibility of using these self-tapping screws as a single-step alternative to current resorbable screw implantation methods.	Low Risk of Bias
Koolen, 2016	Silk pins, machined to specific dimensions, were loaded with BMP-2 by immersion in a solution, followed by in vitro assessment of BMP-2 release in PBS over various time intervals, while in vivo experiments involved implanting BMP-2-loaded silk screws alongside control screws in rats to evaluate the effects over 1, 3, and 6-month periods.	BMP-2 loaded silk screws demonstrated enhanced bone regeneration properties with increased collagen deposition and osteoclast/osteoblast activity compared to pure silk screws in a rat model, indicating their potential efficacy for promoting bone healing over a 6-month period.	Low Risk of Bias

Shi, 2022	Gentamicin-loaded silk-based materials, with varying concentrations of gentamicin loaded into each screw, were prepared using a previously reported method, resulting in rods that were further processed into screws for antibacterial evaluation.	Gentamicin-loaded silk-based screws with 4mg/g of gentamicin were securely affixed in rabbit femurs, displaying rough surfaces and close contact with bone cortex, with histological analysis revealing multinucleated macrophages and new bone formation, alongside favorable biocompatibility and clinical potential over a 3-month period.	Low Risk of Bias
Bottagisio, 2022	After surgically inducing femur osteotomy in rats under general anesthesia, femoral defects were inoculated with saline or MRSE strain, then PSF or AFN-PSF sponges were inserted into the fracture site, with perioperative care, euthanasia after eight weeks, and subsequent micro-CT scans, histological analyses, and microbiological tests to assess bone healing and infection; micro-CT images were obtained to measure bony bridging and bone volume, while microbiological tests were conducted to evaluate bacterial colonization in explanted femurs, followed by histological staining for morphology, inflammation, fracture healing, and signs of osteomyelitis.	Vancomycin-enriched silk fibroin sponges (AFN-PSF) effectively combated MRSE infection, leading to nearly complete fracture healing, new bone formation, and reduced inflammation compared to pure silk fibroin sponges (PSF) in a rat model of MRSE-induced nonunion, suggesting the potential of AFN-PSF for local arthroplasty prophylaxis in high-risk infection patients.	Moderate Risk of Bias
Suryavanshi, 2019	Composite screws were implanted in the subcutaneous pockets of SD rats to assess their biocompatibility. Blood collection, histopathology, and biomechanical testing was conducted to evaluate safety and pull-out strength. Biomechanical testing was performed on synthetic bone blocks to assess the pull-out performance of the screws according to ASTM standards, and ACL reconstruction surgeries were conducted using prototype PCL bone screws to evaluate their suitability for the intended application.	The MSP composite (10% MgO, 20% silk, and 70% PCL) exhibited enhanced tensile properties resembling soft tissues, demonstrating normal organ function and superior pull-out strength compared to PCL alone, suggesting its potential for orthopedic applications.	Low Risk of Bias
Zhang, 2022	Cylindrical hydrogels were implanted subcutaneously in rats for 2, 4, and 8 weeks, followed by histological analysis with hematoxylin and eosin staining. Additionally, a rat model of screw implantation was employed, where self-tapping screws were inserted into the femoral condyle of rats, and analyses including radiographic examination, micro-CT, MRI, toluidine blue staining, and van Gieson staining were conducted at 2–8 weeks post-operation to evaluate bone healing and screw integrity.	They observed preliminary resorption and degradation of both RSF and RSF/G hydrogels, with RSF/G hydrogels showing more degradation at eight weeks, indicating good biocompatibility; both hydrogels maintained screw morphology and demonstrated sufficient strength for insertion into bone, with RSF/G screws exhibiting higher MRI signals, suggesting their potential in orthopedic bone fixation applications.	Moderate Risk of Bias
Yeon, 2017	Femoral bone fractures were surgically induced in 6 Sprague-Dawley rats under general anesthesia and the osteotomy site with a PLA/HA/Silk composite bone clip was fized, followed by evaluation of healing and alignment using CT scans after 8 weeks and sacrifice of the animals at 1, 2, and 4 weeks post-surgery, with subsequent	The PLA/HA/Silk composite bone clip possessed superior biocompatibility and facilitated excellent alignment of bony segments across femur fracture sites compared to PLA and PLA/HA clips, offering an innovative, 3D-printed, patient-specific solution that overcomes limitations of	Low Risk of Bias

	histological analysis of the specimens through hematoxylin and eosin staining.	traditional internal fixation devices and reduces associated complications.	
Yan, 2021	Forty-eight male New Zealand rabbits underwent medial parapatellar arthrotomy and dislocation of the patella to establish an anterior cruciate ligament reconstruction (ACLR) model, with autografts inserted into bone tunnels drilled into the femur and tibia, followed by insertion of silk fibroin (SF) or SF/40% hydroxyapatite (HA)-SF screws; micro-computed tomography scanning and histological analyses were conducted at 4 and 12 weeks postoperatively, and biomechanical analysis was performed to assess maximal failure load, stiffness, range of motion, and site of failure.	The SF/40% HA–SF group exhibited notably higher maximal failure load and stiffness, superior osteoinductivity, and a distinct four-layer structure of the regenerated tendon–bone interface compared to other groups, indicating promising potential for clinical translation of this composite material in rabbit anterior cruciate ligament (ACL) reconstruction.	Low Risk of Bias
